# Probing the surface-localized hyperthermia of gold nanoparticles in a microwave field using polymeric thermometers†
†Electronic supplementary information (ESI) available. See DOI: 10.1039/c5sc01535a


**DOI:** 10.1039/c5sc01535a

**Published:** 2015-07-10

**Authors:** Christopher P. Kabb, R. Nicholas Carmean, Brent S. Sumerlin

**Affiliations:** a George & Josephine Butler Polymer Research Laboratory , Center for Macromolecular Science & Engineering , Department of Chemistry , University of Florida , PO Box 117200 , Gainesville , FL 32611-7200 , USA . Email: sumerlin@chem.ufl.edu

## Abstract

The surface-localized hyperthermia of gold nanoparticles under microwave irradiation was examined. Gold nanoparticles with a hydrodynamic diameter of ∼6 nm stabilized by polymeric “thermometers” were used to gather information on the extent of heating as well as its spatial confinements. Reversible addition–fragmentation chain transfer polymerization was employed to synthesize well-defined, functional polymers of predetermined molecular weights, allowing for estimation of the distance between the nanoparticle surface and the polymer chain end. The polymers were conjugated with a fluorescent dye separated by a thermally-labile azo linkage, and these polymeric ligands were bound to gold nanoparticles *via* gold–thiolate bonds. Conventional heating experiments elucidated the relationship between temperature and the extent of dye release from the gold nanoparticle using fluorescence spectroscopy. The local temperature increase experienced under microwave irradiation was calculated using the same methodology. This approach indicated the temperature near the surface of the nanoparticle was nearly 70 °C higher than the bulk solution temperature, but decreased rapidly with distance, with no noticeable temperature increase when the azo linkage was approximately 2 nm away.

## Abstract 2

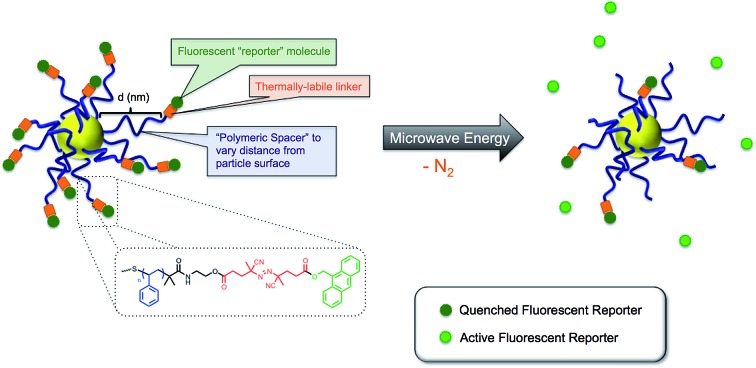
Gold nanoparticles decorated with “polymeric thermometers,” consisting of a polymeric spacer, thermally-labile azo linker, and fluorescent tag, were used to quantify the extent of localized hyperthermia under microwave irradiation.

## Introduction

Localized heating of metal nanoparticles driven by an external stimulus has generated significant interest in recent years, as it provides excellent spatial and temporal control over desired transformations. Theoretical calculations have suggested that appreciable levels of heating are confined to the surface of metal nanoparticles under electromagnetic irradiation,^1^ with the effect rapidly diminishing as distance from the particle is increased. Recent findings by Pellegrino and coworkers, in which decorated iron oxide particles were subjected to an alternating magnetic field (AMF), clearly showed a gradient in which high local temperatures near the surface quickly dissipated as distance increased.^2^ This phenomenon has been exploited to complete a variety of transformations selectively at the nanoparticle surface.

The utility of this heating effect has been demonstrated through the pairing of various nanoparticles and irradiation sources.^3^ Schubert and coworkers subjected iron oxide and nickel nanoparticles to microwave irradiation in the presence of a carbon source to promote the growth of carbon nanofibers.^4^ The authors observed growth exclusively from the surface of the metal nanoparticles and, importantly, nanofiber formation was not observed in the bulk, as the solution temperature remained below the reaction threshold. These results offered indirect evidence of significantly elevated temperatures in the area surrounding the nanoparticles, facilitating nanofiber growth. Additionally, AMF-promoted heating of iron oxide nanoparticles has been extensively studied for use in thermal therapy, in which malignant cancer cells are ablated by the heat generated during irradiation.^5^,^6^ Although iron oxide nanoparticles demonstrate low cytotoxicity and rapid heating *in vivo*, the high concentration of particles required limits the selectivity of the technique, leading to the death of nonmalignant cells surrounding the tumor.

Gold nanoparticles have also been studied for triggered hyperthermia as they are non-toxic, easily synthesized in a wide range of well-defined sizes and shapes, and respond to a variety of stimuli (*e.g.*, visible light, microwave, and radiofrequency irradiation).^7^ An increased surface temperature is experienced in response to light irradiation with a wavelength equal to the surface plasmon resonance (SPR) band (*i.e*., photothermal effect).^8^ This has led to the proposed use of gold nanoparticles as triggered drug delivery systems, in which guest molecules can be released upon sufficient heating generated through photochemical irradiation.^9^ Additionally, this light-driven response has been exploited for controlled deformations of macroscopic shape-memory polymeric nanocomposites.^10^ Moreover, microwave irradiation of gold nanoparticles has also been proposed to result in release of energy, and this phenomenon has led to the use of gold nanoparticles as “nanoscopic radiators”^11^ in which the surface temperature of the gold nanoparticle is selectively increased relative to the bulk solution in a microwave field. Recent examples, including the destruction of osteosarcoma cells^12^ and the disruption of β-amyloid formation,^13^ indicate the potential use of gold nanoparticles and microwave irradiation for thermal ablation in hyperthermia therapy.^3^,^14^–^17^ While the goal of this research is not to investigate systems designed for biological applications, a detailed understanding of localized hyperthermia could facilitate the development of sophisticated nanoparticle systems for a variety of targeted applications.

“Molecular thermometers” have been developed to probe the surface temperature of gold nanoparticle systems under stimuli such as light irradiation and inductive coupling.^18^–^21^ These studies provided insight into nanoparticle-localized heating phenomena, however, the results yielded only indirect evidence, with the magnitude and effective range of heating not having been quantified. Furthermore, such a study on gold nanoparticles has not been conducted in the presence of a microwave field. Increased reaction rates in organic synthesis in a microwave field have been reported.^22^–^29^ So called “microwave-specific” effects have been proposed to describe selective heating of a microwave-absorbing species in a microwave transparent solvent. We expected that heating a solution of gold nanoparticles in a non-polar solvent by microwave irradiation would cause the area directly surrounding the particle to experience a temperature increase relative to the bulk solution. Inspired by the work of Pellegrino *et al.*^2^ that previously allowed the surface temperatures of iron oxide nanoparticles to be measured during exposure to an alternating magnetic field, herein we report a convenient approach to quantify the extent of surface-specific hyperthermia during microwave irradiation of metal nanoparticles in solution. This method allowed us to gather spatial information on temperature (*i.e.*, temperature gradient) at the subnanometer level during microwave irradiation.

Three main components were necessary: (1) a well-defined polymer of controlled molecular weight (*i.e*., polymeric spacer) capable of durable attachment to the gold nanoparticle surface; (2) a fluorescent tag; and (3) a thermally-labile linkage between the distal terminus of the polymeric spacer and the fluorescent tag. Using this approach, gold nanoparticles were decorated with thermally-labile fluorescent tags at varying distances from the particle surface determined by the molecular weight of the polymer spacer. We reasoned that under microwave irradiation, the azo linkages would degrade to release the fluorescent tag into solution, with the rate of release being directly correlated to the temperature in the vicinity of the azo linkage. Therefore, by varying the length of the polymer spacer, information was obtained on the gradient of temperature from the particle surface to the bulk solution. To our knowledge, this is the first direct experimental measurement of microwave-induced hyperthermia of metal nanoparticles.

## Results and discussion

### Synthesis of polymeric spacers

To determine the surface-specific heating of nanoparticles in microwave field, it was important to choose a polymer and solvent system that were relatively microwave-transparent (*i.e*., nonpolar) to reduce undesired bulk heating events^30^ and allow accurate measurement of the release kinetics of the fluorescent tag. For this reason, nonpolar polystyrene (PS) was chosen as the polymeric spacer and toluene as the solvent. These two components are essentially microwave transparent, while the dielectric loss tangent (tan *δ*) of gold is significantly higher.^31^,^32^ Therefore, we reasoned that microwave irradiation of a solution of PS-coated gold nanoparticles would result in selective absorbance by the metal nanoparticles, producing free electrons and phonons, and lead to subsequent dissipation as thermal energy.^33^,^34^

**Scheme 1:**
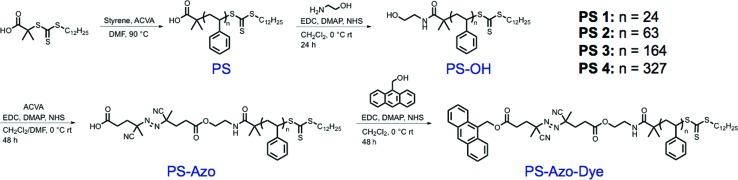
Synthesis of functionalized polymeric spacers. Sequential EDC couplings of polystyrene (PS) with ethanolamine, 4,4′-azobis(4-cyanovaleric acid) (ACVA), and 9-anthracenemethanol yielded “polymeric thermometers” to be used for temperature measurement on the surface of gold nanoparticles.

Polystyrene was prepared *via* reversible addition–fragmentation chain transfer (RAFT) polymerization to yield polymers of pre-determined molecular weights and narrow molecular weight distributions (Table 1). The well-defined nature of polymers synthesized in this manner allowed for precise control over the distance of the thermally-labile linker from the nanoparticle surface. Furthermore, the use of a RAFT chain transfer agent allowed for inclusion of functional end-groups, including a terminal trithiocarbonate moiety that was readily reduced to yield thiol-terminated polymers capable of durable attachment to the gold nanoparticle surface. The carboxy terminus of the polymers was treated with ethanolamine to yield hydroxyl-terminated polymers (PS–OH) capable of further functionalization. Generally, thiocarbonylthio groups on RAFT-generated polymers undergo aminolysis in the presence of a nucleophilic amine.^35^,^36^ However, careful addition of a small excess of ethanolamine at 0 °C under EDC/NHS coupling conditions led exclusively to reaction with the activated ester.^37^,^38^ Gel permeation chromatography (GPC) did not show evidence of disulfide formation, which would be expected if aminolysis was appreciable (Fig. S1a†). Furthermore, a lack of aminolysis of the trithiocarbonate was confirmed through comparison of the UV-Vis spectra of the starting material and product at 305 nm (Fig. S1b†). A thermally labile azo initiator, 4,4′-azobis(4-cyanovaleric acid), and fluorescent reporter molecule, 9-anthracenemethanol, were conjugated to the macromolecule *via* sequential EDC coupling reactions to afford the functionalized polymeric spacers, **PS-Azo-Dye 1–4** (Scheme 1). Attachment of the anthracene dye to the polymer chain ends was verified using UV-Vis, fluorescence, and ^1^H NMR spectroscopy (Fig. S2–S4†).

**Table 1:** Properties of polystyrene spacers **PS 1–4**

Entry	DP_n_^*a*^	*M*_n_ ^*a*^ (g mol^–1^)	*M*_w_ ^*a*^ (g mol^–1^)	*M*_w_/*M*_n_^*a*^	*R*_g_ ^*b*^ (nm)
**PS 1**	24	2 500	2 600	1.05	0.51
**PS 2**	63	6 600	6 700	1.01	0.83
**PS 3**	164	17 100	17 800	1.04	1.34
**PS 4**	327	32 100	34 400	1.07	1.87

^*a*^Determined from GPC-MALLS.

^*b*^Calculated using eqn (1).

Previous reports of conformational analysis of polymers docked to a gold surface have suggested that the chains primarily exist in a random coil conformation, provided that a good solvent for the polymer is used and there is minimal interaction between the repeat units and the surface.^39^,^40^ Toluene is a good solvent for polystyrene,^41^ thus we assumed that the treatment of the polymers as random coils was appropriate in our system. Furthermore, variations in chain conformation due to the dynamic conditions experienced in our study are expected to have only a limited effect on the observed heating profiles. Therefore, it was also expected that the average radius of gyration (*R*_g_) of the polymer would allow for an estimation of the effective distance between the degradable azo-containing chain end and the particle surface.

For polymers attached to nanoparticles, eqn (1) (ref. 42) approximates the radius of gyration in a random coil conformation:1
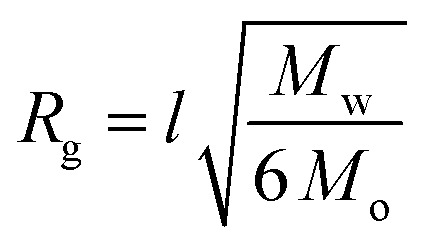
where *l* is the contour length of a single styrenic repeat unit (0.252 nm),^43^*M*_w_ is the weight-average molecular weight of the polymer, and *M*_o_ is the repeat unit molecular weight (104.15 g mol^–1^) (Table 1).

### Gold nanoparticle synthesis

**Fig. 1:**
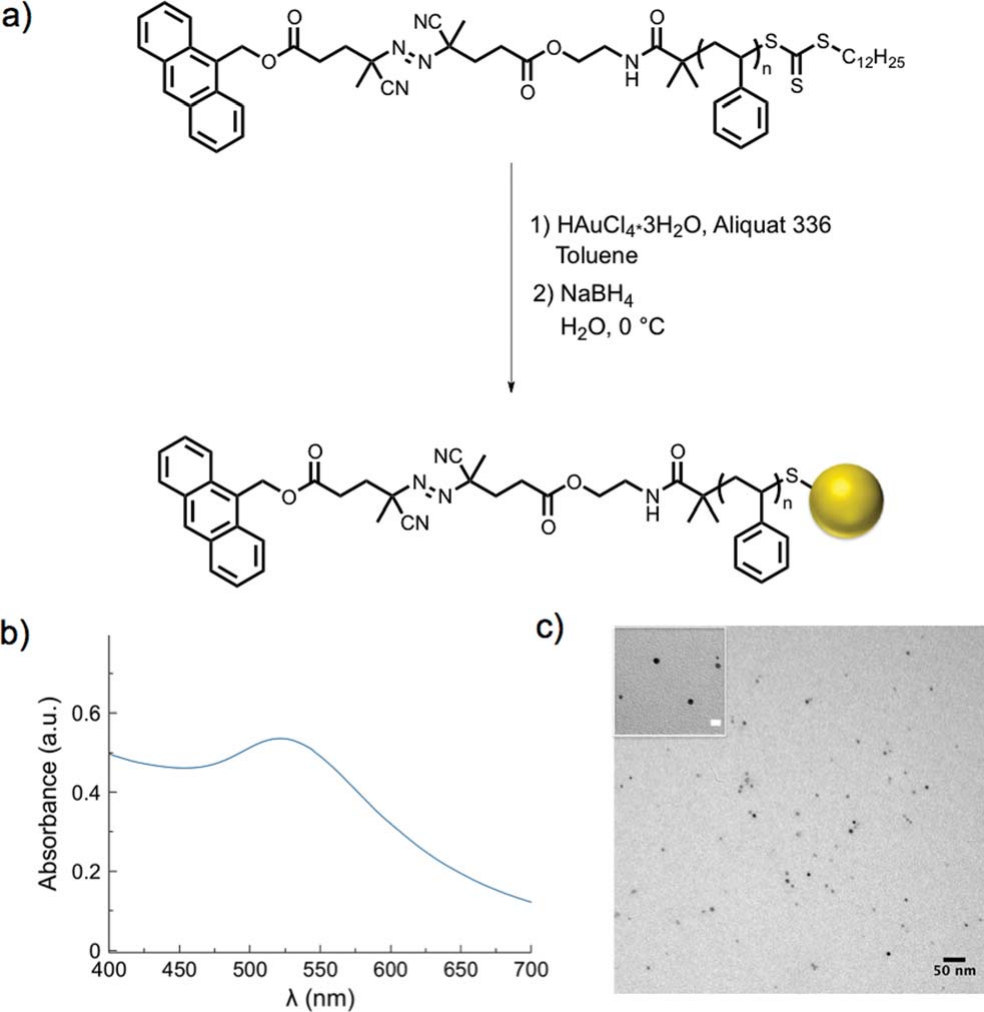
(a) Synthesis of the polymer-modified gold nanoparticles *via* one pot phase transfer synthesis; (b) UV-Vis spectrum displaying characteristic SPR absorbance with *λ*_max_ = 520 nm; (c) representative TEM micrograph of gold nanoparticles (inset scale bar is 10 nm).

Gold nanoparticle synthesis typically requires elevated temperatures^44^ or a strong reducing agent (*e.g.*, superhydride)^45^ at lower temperatures. However, in our case, high temperatures would result in premature dissociation of the azo moieties and a strong reducing agent could likely cleave either of the two esters on the α-chain end of the polymer. Both of these strategies would result in loss of the fluorescent tag from the polymeric ligands. Therefore, a modified two-phase nanoparticle synthesis was carried out at 0 °C.^46^,^47^ A solution of **PS-Azo-Dye**, chloroauric acid (HAuCl_4_·3H_2_O), and Aliquat 336 (a phase-transfer catalyst) in toluene was added to an ice-cold solution of sodium borohydride. As the gold salt was reduced and began to form colloidal gold, the trithiocarbonate moiety of the polystyrene was simultaneously reduced, resulting in stabilized thiolate-protected gold nanoparticles.^48^–^50^ A change in color of the organic solution from yellow to deep red indicated the rapid formation of gold nanoparticles, and after two hours, narrowly dispersed gold nanoparticles with average diameters in the range of 6 nm were formed, as determined by transmission electron microscopy (TEM, Table 2, Fig. S5†). The SPR *λ*_max_ at 520 nm was also characteristic of gold nanoparticles in this size range^51^ (Fig. 1 and S6†). Following purification *via* repeated centrifugation–decantation cycles, ^1^H NMR spectroscopy of the nanoparticles revealed the presence of polystyrene ligands bound to the nanoparticle surface (Fig. S7†).

**Table 2:** Gold nanoparticle properties

Entry^*a*^	Polymer *M*_w_^*b*^ (g mol^–1^)	*D*_avg_ ^*c*^ (nm)	Ligand density^*d*^ (chains nm^–2^)
**AuNP 1**	2 600	5.7 ± 0.9	0.85
**AuNP 2**	6 700	5.6 ± 0.6	0.28
**AuNP 3**	17 800	5.7 ± 1.1	0.29
**AuNP 4**	34 400	6.0 ± 0.7	0.32

^*a*^Synthesized using **PS-Azo-Dye X** as ligand (X = corresponding polystyrene sample from Table 1).

^*b*^Determined from GPC-MALLS.

^*c*^Average nanoparticle diameter measured from TEM micrographs.

^*d*^Determined by thermogravimetric analysis of “blank” AuNPs.

Polystyrene-functionalized gold nanoparticles were examined by thermogravimetric analysis to approximate the number of stabilizing ligands per nanoparticle area (Fig. S8, Tables 2 and S1†). For example, in the case of **AuNP 3**, the average particle diameter (5.5 ± 0.8 nm), as measured by TEM, was used to calculate the particle volume with the assumption that the particles are spherically shaped 
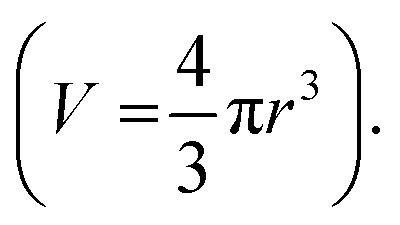
 Using the ideal bulk density of gold^52^,^53^ (19.3 g cm^–3^), the average mass of a single gold nanoparticle was calculated as 1.68 × 10^–18^ g. The percent weight loss of polystyrene from 300–650 °C was used to calculate the number of polymer chains in the sample. The mass remaining at 650 °C was attributed to the gold nanoparticles, and used to determine the number of nanoparticles present. The quotient of the two yielded an average ligand density of 0.29 chains nm^–2^. Through similar analytical methods, Lennox *et al.* calculated a grafting density of 0.94 chains nm^–2^ (polystyrene *M*_n_ ∼ 13 kg mol^–1^). The increased grafting density relative to our measurement may be attributed to the different method of gold nanoparticle preparation chosen. Importantly, Lennox *et al.* also determined this grafting density resulted in chain conformations between extended and random-coil.^54^ The lower grafting density observed in our system should lead to less significant interactions between neighboring chains and an increased probability of conformations that approach those of a random coil, thereby allowing the radius of gyration to provide an estimation of the distance of the end-tethered fluorescent tag from the nanoparticle surface.

The thermal sensitivity of the azo moiety on the polymeric spacer made it difficult to completely dry the nanoparticles (which would require heating) to accurately measure their concentration. To determine the concentration of functionalized gold nanoparticles in solution, a set of “blank” nanoparticles stabilized by **PS 1–4** without the azo-linked fluorophore was synthesized. These particles were stable to high temperature and low pressure, allowing them to be dried completely and weighed. A calibration curve was prepared using these solutions by absorbance measurements of the SPR band (Fig. S9†). The linear fits of these calibration plots were used to calculate the concentration of fluorescently-tagged gold nanoparticles (**AuNP 1–4**) in their respective solutions. Stock solutions of these gold nanoparticles in toluene (0.2 mg mL^–1^) were prepared and used for all further measurements.

### Conventional heating calibrations

The fluorescence quenching effect of gold nanoparticles has been extensively studied.^55^–^57^ It has been reported that up to 99.8% of fluorescence intensity is quenched at a distance of 1 nm from the surface,^55^ and significant quenching is still observed up to 8 nm away.^57^ Therefore, fluorescent tags attached to the nanoparticle surface are expected to be quenched and only fluoresce upon release from the polymeric ligand into solution. This property allows us to use gold nanoparticles as a fluorescence “switch”, turning fluorescence “off” when the ligands are bound and “on” upon their cleavage and subsequent dissociation. To determine if the gold nanoparticles could quench fluorescence of the tag after it was released, a dilute solution of 9-anthracenemethanol (10 μg mL^–1^) was prepared, and fluorescence was measured in the presence and absence of gold nanoparticles (0.2 mg mL^–1^, Fig. S10†). Due to the markedly low concentration of nanoparticles in solution, the probability of interaction with 9-anthracenemethanol was negligible. Therefore, a quenching effect was not observed, and it was determined that removal of nanoparticles was not necessary prior to fluorescence measurements following heating.

**Fig. 2:**
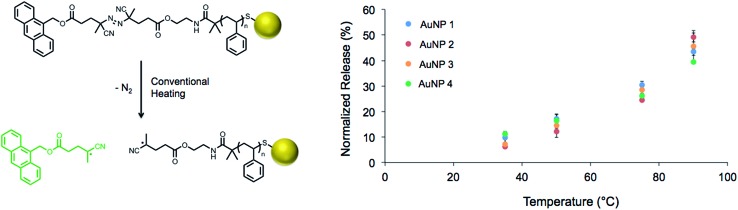
Thermal dissociation of the fluorescent tag during conventional heating of the polymer-modified nanoparticles for 1 h (left); calibration for release of the fluorescent tag from the gold nanoparticles stabilized by **PS-Azo-Dye 1–4** (right; see Table 2 for specific information on **AuNP 1–4**).

Solutions of decorated gold nanoparticles (0.2 mg mL^–1^) were exposed to a pre-heated oil bath at various temperatures ranging from 35 to 90 °C for 1 h, and the increase in fluorescence of each solution was measured immediately thereafter (Fig. S11†). These measurements were normalized to the complete release of the anthracene segment (90 °C for 18 h, Fig. S12†), and the extent of release showed a strong dependence on temperature (Fig. 2). It is important to note that the rate of release was completely independent of spacer length, as would be expected due to the homogeneous heating of the nanoparticle solution under these conventional heating conditions. Additionally, the similar levels of fluorescence observed at each temperature suggested the conjugation efficiency was similar across the samples (**AuNP 1–4**) and was not affected by the polymer molecular weight.

### Microwave irradiation of gold nanoparticles

The decorated nanoparticles in toluene were exposed to microwave irradiation for 1 h. Immediately following irradiation, the bulk solution temperature (*T*_bulk_) was measured using an external thermocouple before cooling in an ice-water bath to prevent further release of the fluorescent tag. The change in fluorescence of the gold nanoparticle solution following irradiation was correlated to the respective conventional heating calibration curve using an exponential fitting equation (eqn (2a) and (2b)) to provide an approximate value for the apparent temperature (*T*_app_) to which the azo-linked fluorophore was exposed:

2a *I*_max_ = *A* e^(*T*/*τ*)^

2b
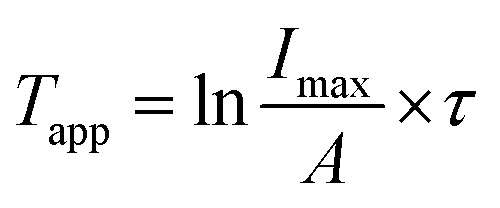
where *I*_max_ is the normalized fluorescence intensity at 415 nm, *T* is the applied temperature in conventional heating, and *τ* is the decay rate.

Assuming the rate of azo-moiety dissociation was only dependent on temperature and not on the mode of heating (*i.e*., conventional *vs.* microwave),^58^,^59^ *T*_app_ is an approximation of the temperature at the average distance of the fluorescent tag from the nanoparticle surface. Comparing *T*_app_ to *T*_bulk_ using eqn (3) allows the increased local temperature attributed to microwave irradiation of the gold particles (Δ*T*_local_) to be determined.

3 Δ*T*_local_ = *T*_app_ – *T*_bulk_

**Fig. 3:**
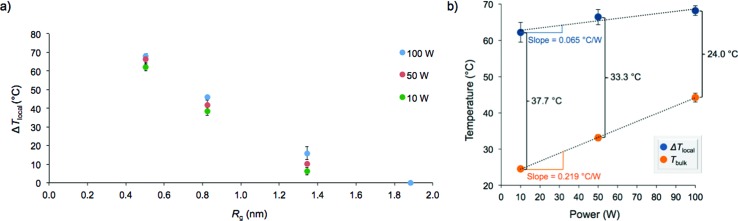
Microwave heating of functionalized gold nanoparticles at 10 W, 50 W and 100 W for 1 h. (a) Change in local temperature (Δ*T*_local_) as a function of the radius of gyration (*R*_g_) of the stabilizing polymer; (b) changes in local temperature and bulk temperature (*T*_bulk_) for **AuNP 1** as a function of microwave power.

If surface-specific heating was operable, Δ*T*_local_ should decrease with increasing distance from the nanoparticle surface. Therefore, the obtained values of Δ*T*_local_ for **AuNP 1–4** were plotted as a function of the radius of gyration of the stabilizing polymers (Fig. 3a). Indeed, the extent of localized hyperthermia showed a strong dependence on the distance between the azo moiety and the nanoparticle surface. In close proximity to the nanoparticle, the temperature increase reached nearly +70 °C, while essentially no localized hyperthermia was observed approximately 2 nm from the particle surface. Under the dilute conditions employed, we postulated that degradation of the azo moiety and subsequent release of the fluorescent tag into solution was primarily due to localized heating of the particles to which the fluorophores were bound, rather than the heat generated by neighboring nanoparticles. This was confirmed by monitoring the extent of fluorophore release as a function of concentration. We noted that the change in fluorescence intensity following irradiation (100 W) of a solution of nanoparticles at a concentration of 0.1 mg mL^–1^ was proportional to the fluorescence intensity observed at 0.2 mg mL^–1^ (*i.e.*, 156 ± 24 *vs.* 292 ± 12, respectively). If interactions between neighboring particles were operative in this concentration range, the change in fluorescence intensity would not be expected to be proportional to the change in concentration.

The effect of microwave power on the extent of heating was also examined by monitoring release of the fluorescent tag at 10, 50, and 100 W (attempts at 1 W led to inconsistent temperature measurements and significant error). The local temperature increase was not drastically dependent on microwave power above 10 W, and as a result, high surface-localized temperatures were still attainable at low microwave power (Fig. 3b and S13†). However, the bulk solution temperature was much more significantly affected by microwave power, increasing at a rate greater than three times that of Δ*T*_local_. This is an important finding since employing lower microwave power reduces background bulk heating during irradiation while still allowing for elevated temperatures near the surface of the nanoparticle. These results could be useful synthetically to potentially enhance the efficiency and/or selectivity of surface-catalyzed reactions at relatively lower temperatures.

To ensure *T*_bulk_ measured after 1 h was representative of the entire irradiation time, solutions of gold nanoparticles in toluene were irradiated at varying powers, and *T*_bulk_ was measured in ten-minute intervals using an external thermocouple (Fig. S14†). The solutions heated rapidly to their maximum temperature, and remained constant throughout the rest of the irradiation time. Furthermore, a kinetic study was carried out to determine the extent of fluorophore release as a function of time. Compared to conventional heating, a similar release profile is observed, and the extent of microwave-induced release agrees closely with those samples heated in an oil bath (Fig. S15†). Moreover, the exponential decay of the plot indicates a consistent temperature throughout the reaction, rather than one that is constantly increasing. These results provide evidence that *T*_app_ measured after 1 h is representative of the temperature during the entirety of the irradiation period.

Interestingly, after extended storage of gold nanoparticles (>3 months), the local temperature (*T*_app_) observed during microwave irradiation was significantly higher than the values previous observed for freshly prepared nanoparticle solutions. TEM indicated that aggregation of the nanoparticles had occurred during storage. After sonicating these samples, however, the aggregates were disrupted, and a uniform dispersion of nanoparticles was observed by TEM (Fig. S16†). Microwave heating of the sonicated samples resulted in apparent local temperatures that were nearly identical to the values obtained immediately after nanoparticle synthesis (*i.e*., prior to storage). Therefore, this rise in apparent local temperature was attributed to the aggregation of nanoparticles over time leading to heating of azo linkages by multiple adjacent particles, or to the effect of nanoparticle size on microwave-induced surface-specific heating.

**Fig. 4:**
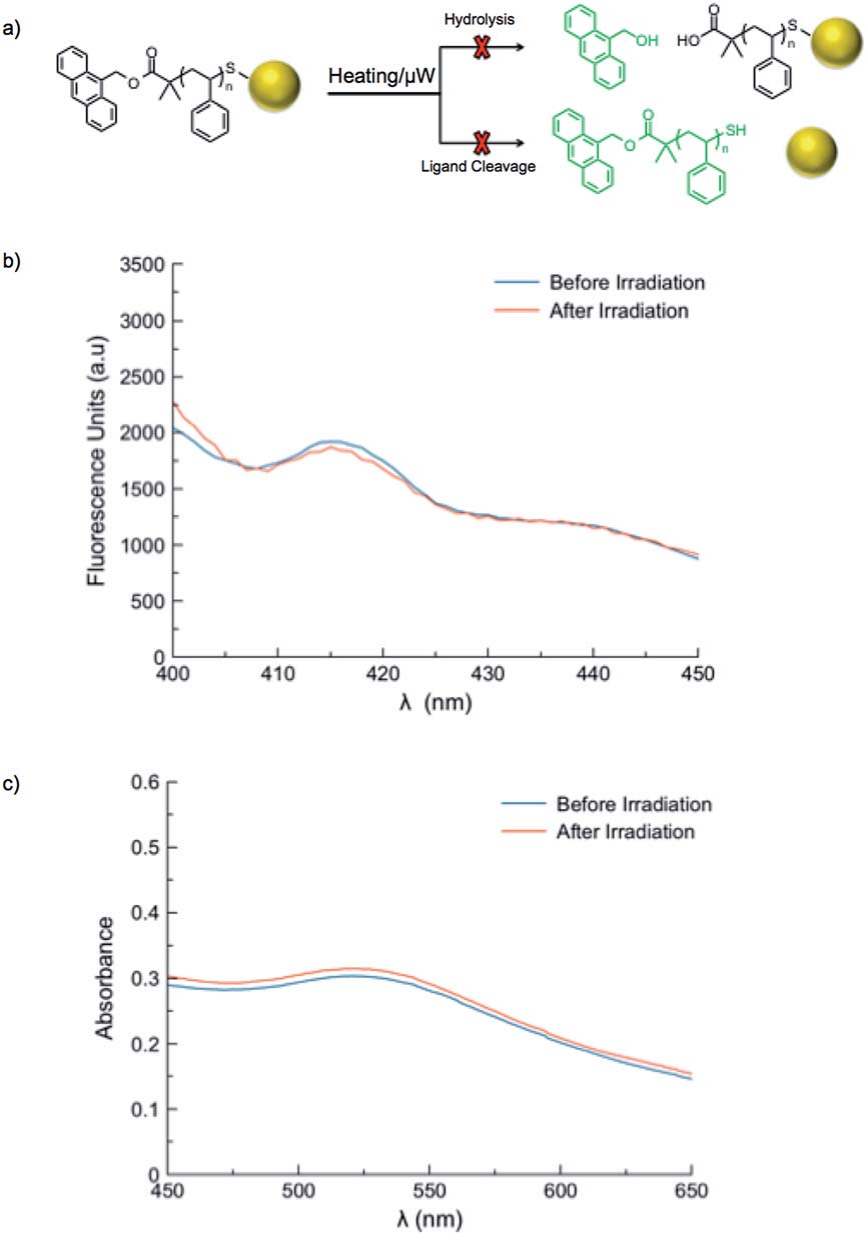
Microwave control reactions (100 W) confirmed that the fluorescent dye was not released from gold nanoparticles stabilized with polymer that did not contain the thermally-labile azo linkage. (a) Schematic representation of possible alternate routes of fluorescent dye release from gold nanoparticles during control studies; (b) fluorescence emission spectra before and after microwave irradiation (*λ*_ex_ = 360 nm). No increase in the fluorescence intensity is observed following irradiation; (c) UV-Vis absorbance spectra of gold nanoparticles prior to and following microwave irradiation. The *λ*_max_ remains at 520 nm, indicating the size and shape of the nanoparticles are consistent pre- and post-irradiation.

A series of control reactions were carried out to confirm the mechanism of fluorescence release was solely based upon cleavage of the azo moiety, rather than ester hydrolysis or cleavage of the ligands from gold nanoparticles (Fig. 4a). A sample of polystyrene directly functionalized with 9-anthracenemethanol (**PS-Dye**, no azo linker) was used to prepare gold nanoparticles. Upon heating at 90 °C for one hour, no change in fluorescence was observed. Likewise, exposure to microwave irradiation (100 W for 1 h) resulted in no release of the anthracene tag (Fig. 4b). If the ester linkages were to degrade upon exposure to microwaves or heat, an increase in fluorescence would be expected. Furthermore, the SPR *λ*_max_ remained unchanged after microwave irradiation or conventional heating, suggesting the size and shape of the gold nanoparticles remained consistent (Fig. 4c). Although the reported stability of gold–thiol interactions widely varies,^21^,^60^–^62^ our results suggest adequate thermal stability in our system and indicate the change in fluorescence observed for **AuNP 1–4** is due to cleavage of the thermally-labile azo linker, rather than destruction of the thiolate–gold interactions.^9^,^63^

## Conclusions

In summary, a system was developed to study the localized heating effects at the surface of gold nanoparticles irradiated with microwave energy. The release of a fluorescent dye from a thermally-labile azo linkage was monitored to determine the extent of heating, while the use of well-defined polymeric spacers allowed for tuning of the distance between the particle surface and the azo moiety. The particles were exposed to microwave irradiation, and while the bulk temperatures remained low, it was determined that the heating effect was significant at short distances from the surface (Δ*T*_local_ ~ +70 °C at *R*_g_ = 0.51 nm) and rapidly diminished as distance from the particle increased (no apparent temperature increase at *R*_g_ = 1.87 nm). Dependence of the local temperature increase on microwave power is much less pronounced, with differences of only 6–8 °C occurring upon increasing the power from 10 to 100 W.

To our knowledge, this is the first example of the direct measurement of microwave-induced hyperthermia of metal nanoparticles in solution. Not only does this approach demonstrate that surface-specific heating occurs, but also it also provides insight into the magnitude of hyperthermia as a function of radial distance from the particle surface. Studies on the effect of nanoparticle size on the extent of localized heating may elucidate the mechanism for this temperature increase. A detailed understanding of localized hyperthermia could facilitate the development of more sophisticated nanoparticle systems for targeted and delivery applications. Moreover, the capabilities offered by this mode of heating may aid in conducting organic reactions with increased spatial control. In principle, the versatility of the synthetic approach employed to prepare the polymeric ligands should allow this general strategy to be exploited for any nanomaterial that responds to microwaves by emitting heat, including nanoparticles of other metals, magnetic nanoparticles, carbon nanotubes, *etc.*

## Supplementary Material

Supplementary information
