# Supplementary material for: Probing the surface-localized hyperthermia of gold nanoparticles in a microwave field using polymeric thermometers
Source: Chem Sci. 2015 Jul 10;6(10):5662–9. doi: 10.1039/c5sc01535a (PMC5949850; doi:10.1039/c5sc01535a)
Supplement: Supplementary file 1 [file SC-006-C5SC01535A-s001.pdf]

Electronic Supplementary Material (ESI) for Chemical Science.
This journal is © The Royal Society of Chemistry 2015

Supplementary Information

For

# Probing the surface-localized hyperthermia of gold nanoparticles in a microwave field using polymeric thermometers

Christopher P. Kabb, R. Nicholas Carmean and Brent S. Sumerlin*

George & Josephine Butler Polymer Research Laboratory, Center for Macromolecular Science & Engineering, Department of Chemistry, University of Florida, PO Box 117200, Gainesville, FL 32611-7200, USA. E-mail: sumerlin@chem.ufl.edu; Fax: +1 352 392 9741

## Materials

All chemicals were used as received unless otherwise noted. 2-(Dodecylthiocarbonothioylthio)-2-methylpropionic acid was synthesized according to a previous report.[55] Styrene was passed through a column of basic alumina to remove inhibitors and acidic impurities prior to polymerization. *N*-Hydroxysuccinimide (NHS, 98%, Sigma-Aldrich) was recrystallized from ethanol. Ethanolamine (99.5%) was purchased from VWR. 4,4'-Azobis(4-cyanovaleric acid) (ACVA, 98%), 9-anthracenemethanol (9AM, 97%), chloroauric acid ($HAuCl_4$•$3H_2O$, >99.9%), toluene (99.8%), *N*,*N*-dimethylformamide (DMF, >99%) and sodium borohydride (>98.5%) were purchased from Sigma-Aldrich. 1-Ethyl-3-(3-dimethylaminopropyl)carbodiimide hydrochloride (EDC, >98%) was purchased from TCI. 4-Dimethylaminopyridine (DMAP, 98%) was purchased from Acros Organics. Aliquat 336 was purchased from Alfa Aesar.

## Experimental

**RAFT polymerization of styrene.** In a typical synthesis of **PS 4**, styrene (5.40 g, 51.8 mmol), 2-(dodecylthiocarbonothioylthio)-2-methylpropionic acid (25.0 mg, 68.7 µmol), ACVA (2.1 mg, 7.5 µmol), and DMF (10.4 mL) were charged to a 50 mL Schlenk flask equipped with magnetic stirrer. The solution was degassed by three successive freeze-pump-thaw cycles, filled with nitrogen, and subjected to an oil bath at 80 °C. Reaction progress was monitored by GPC and quenched at the desired molecular weight. The reaction mixture was precipitated (×3) into cold methanol, and the resulting yellow powder was dried in vacuo.

**Synthesis of PS-OH. PS 4** (1.0 g, 28 µmol), EDC (6.6 mg, 34 µmol), NHS (6.6 mg, 57 µmol and DMAP (4.2 mg, 34 µmol) were stirred in anhydrous DCM (10 mL) for 60 min. The reaction mixture was cooled to 0 °C, and ethanolamine (1.9 mg, 32 µmol) in anhydrous DCM (1 mL) was added drop-wise over 45 min. The reaction was allowed to warm to room temperature and stirred for 24 h. The solution was washed with DI water (3 × 100 mL) and brine (2 × 100 mL) and dried over $MgSO_4$. The mixture was filtered, concentrated by rotary evaporator, and dried in vacuo to afford **PS-OH 4** as a yellow powder (0.76 g, 75.9%).

**Synthesis of PS-Azo.** ACVA (30 mg, 0.11 mmol), EDC (10 mg, 54 µmol), NHS (6.2 mg, 54 µmol) and DMAP (6.6 mg, 54 µmol) were stirred in anhydrous DMF (1 mL) for 60 min. The reaction mixture was cooled to 0 °C, and **PS-OH 4** (0.76 g, 26 µmol) in anhydrous DCM (6 mL) was added drop-wise over 45 min. The reaction was allowed to warm to room temperature and stirred for 48 h in the dark. The solution was washed with DI water (3 × 100 mL) and brine (2 × 100 mL) and dried over $MgSO_4$. The mixture was filtered, concentrated by rotary evaporator, and dried in vacuo to afford **PS-Azo 4** (0.53 g, 58.1%).

**Synthesis of PS-Azo-Dye. PS-Azo 4** (0.53 g, 16 µmol), EDC (3.5 mg, 18 µmol), NHS (4.4 mg, 38 µmol), and DMAP (2.2 mg, 18 µmol) were stirred in anhydrous DCM (2.5 mL) for 60 min. The reaction mixture was cooled to 0 °C, and 9-anthracenemethanol (7.9 mg, 38 µmol) in anhydrous DCM (2.5 mL) was added drop-wise over 45 min. The reaction was allowed to warm to room temperature and stirred for 48 h. The solution was washed with DI water (3 × 100 mL) and brine (2 × 100 mL) and dried over $MgSO_4$. The mixture was filtered, concentrated by

rotary evaporator, and precipitated (×3) into cold methanol to remove residual 9-anthracenemethanol. The resulting powder was dried in vacuo to afford **PS-Azo-Dye 4** (0.21 g, 39.4%).

**Synthesis of polystyrene-stabilized AuNPs.** To a freshly prepared aqueous solution of sodium borohydride (13.8 mg in 10 mL), a yellow solution of $HAuCl_4$•$3H_2O$ (14 mg, 36 μmol), Aliquat 336 (18 mg, 45 μmol), and **PS-Azo-Dye 4** (0.23 g, 7.2 μmol) in toluene (2.5 mL) was added drop-wise at 0 °C. Upon addition, the organic phase turned a deep ruby red, and the mixture was allowed to stir for an additional 2 h at 0 °C. The organic phase was diluted with toluene (10 mL), washed with DI water (2 × 20 mL) and brine (1 × 20 mL) and dried over $MgSO_4$. The mixture was filtered, and the concentration of nanoparticles in solution was measured by UV-Vis with the use of a standard curve.

**Conventional heating calibration experiments.** A 200 μL aliquot of a solution of **AuNP X (X = 1-4,** 200 μg/mL) in toluene was sealed in a round bottom flask and placed in a temperature controlled oil bath at temperatures ranging from 35-90 °C. After 1 h, the reaction was quenched by immersion in an ice water bath, and fluorescence measurements were taken immediately thereafter. An identical volume (150 μL) of the heated **AuNP X** solution was referenced to an unheated solution and the change in fluorescence was recorded.

**Microwave irradiation of AuNPs.** A 200 μL aliquot of a solution of **AuNP X (X = 1-4,** 200 μg/mL) in toluene was sealed in a microwave vessel and irradiated with fixed power (10 W, 50 W or 100 W) for 1 h. The bulk solution temperature was measured immediately after stopping the reaction with an external thermocouple, the reaction was quenched by immersion in an ice water bath, and fluorescence measurements were taken immediately thereafter. An identical volume (150 μL) of the irradiated **AuNP X** solution was referenced to a solution that was not irradiated and the change in fluorescence was recorded.

**Control experiments.** AuNPs stabilized by **PS-Dye** (no azo moiety) were subjected to conventional heating at 90 °C for 24 h or microwave irradiation at 100 W for 1 h, before quenching by immersion in an ice water bath. No change in fluorescence was observed. To test whether or not the presence of AuNPs in solution would affect the fluorescence measurements of free 9-anthracenemethanol, a solution of AuNPs (0.2 mg/mL) and 9-anthracenemethanol (10 μg/mL) was prepared, and the fluorescence reading was compared to that of a solution of 9-anthracenemethanol in the absence of gold nanoparticles. No noticeable difference was observed, and it was determined that the concentration of nanoparticles in solution was sufficiently low as to not impact the fluorescence readings.

## Instrumentation

**Microwave Irradiation.** All microwave irradiation experiments were conducted in 10 mL glass microwave vessels on a CEM Discover SP microwave reactor ($f$ = 2.45 GHz) operating in Fixed Power mode. Compressed air (20 PSI) was passed continuously over the exterior surface of the tube to limit heating of the glass vessel.

**Nuclear Magnetic Resonance (NMR) Spectroscopy.** $^1$H NMR spectra were recorded in $CDCl_3$ using an Inova 500 MHz spectrometer.

**Gel Permeation Chromatography (GPC)**. Molecular weight and polydispersity were determined by gel permeation chromatography in *N*,*N*-dimethylacetamide (DMAc) with 50 mM LiCl at 50 °C and a flow rate of 1.0 mL min$^{-1}$ (Agilent isocratic pump, degasser, and autosampler, columns: PLgel 5 μm guard + two ViscoGel I-series G3078 mixed bed columns: molecular weight range 0−20 × $10^3$ and 0−100 × $10^4$ g mol$^{-1}$). Detection consisted of a Wyatt Optilab T-rEX refractive index detector operating at 658 nm and a Wyatt miniDAWN Treos light scattering detector operating at 659 nm. Absolute molecular weights and polydispersities were calculated using the Wyatt ASTRA software and the *dn/dc* for polystyrene (0.144 mL/g).

**UV-Vis and Fluorescence Spectroscopy.** All measurements were performed on a Molecular Devices SpectraMax M2 Multimode Microplate Reader at 25 °C. Fluorescence measurements were conducted with 150 μL of sample on black 96-well polypropylene microplates (Greiner Bio-One) with an excitation wavelength of 360 nm and $\lambda_{max}^{em}$ = 415 nm. Absorbance measurements were conducted with 150 μL of sample on clear 96-well microplates (Greiner). Calibration curves were constructed using the integrated SoftMax Pro software.

**Transmission Electron Microscopy (TEM)**. A solution of the sample (5 μL) was applied onto a formvar coated 200-mesh Cu grid (Electron Microscopy Sciences) that was freshly glow discharged (Pelco easiGlow™, Ted Pella, Inc.). The grids were observed on a Hitachi H7000 microscope operating at 100 kV. The images were recorded with a slow-scan CCD camera (Veleta 2k × 2k) and iTEM software.

**Thermogravimetric Analysis (TGA).** Thermogravimetric analyses were performed under nitrogen with a TGA Q5000 (TA Instruments). Approximately 1-2.5 mg of gold nanoparticle sample was dissolved in dichloromethane and transferred dropwise to the platinum TGA pan. The pan was dried by blowing nitrogen gas over the pan to evaporate solvent and allow for further dropwise addition of AuNP solution. The samples were heated from 20 °C to 200 °C at a ramp rate of 20 °C $min^{-1}$ to remove moisture. Then, the temperature was returned to 20 °C and samples were heated again at the same ramp rate to 600 °C.

**Supporting Figures**

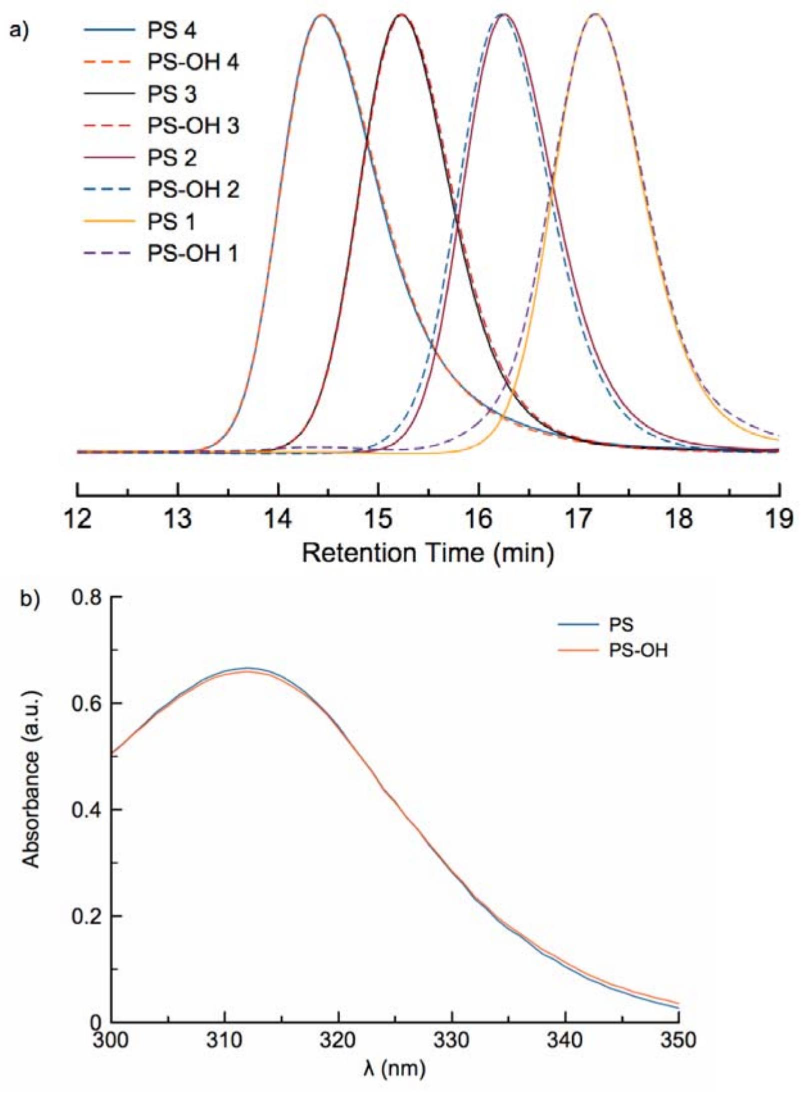


**Fig. S1** Gel permeation chromatography and UV-Vis spectroscopy confirm the retention of the trithiocarbonate moiety following addition of ethanolamine to **PS**. (a) Gel permeation chromatograms show no evidence of disulfide formation, which would be expected if aminolysis of trithiocarbonate moieties to thiols had occurred; (b) The UV-Vis spectra clearly show that the trithiocarbonate moiety is retained, as the absorbance remains consistent following coupling with ethanolamine.

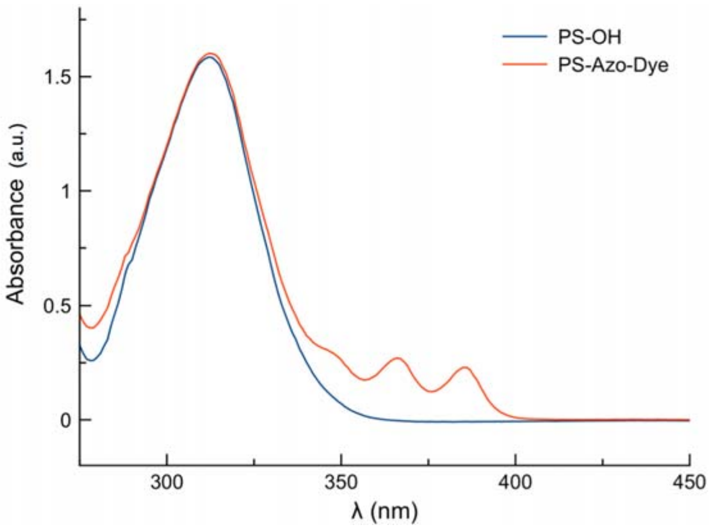


**Fig. S2** UV-Vis spectra of **PS-OH 1** (blue) and **PS-Azo-Dye 1** (orange) reveal the appearance of the absorption band of anthracene from 340-400 nm, confirming the presence of the fluorescent tag on the ligand's terminus.

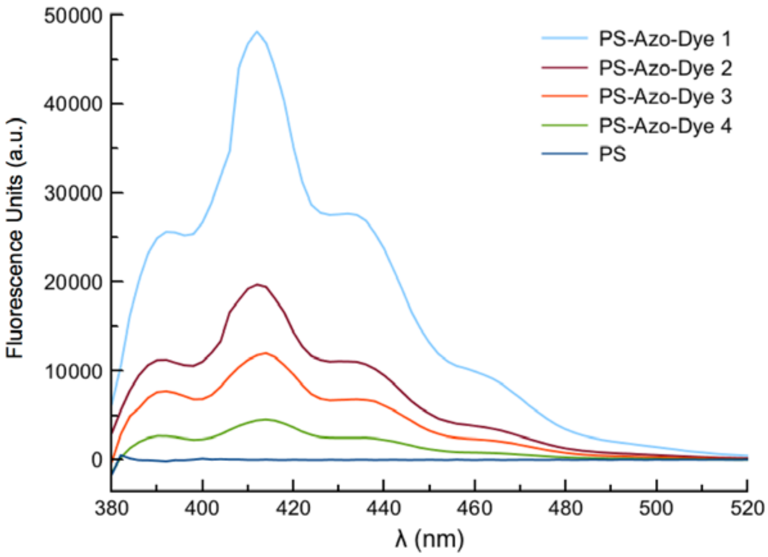


**Fig. S3** Fluorescence spectra ($\lambda^{ex}$ = 360 nm) of functionalized **PS-Azo-Dye** polymeric thermometers (1 mg/mL in THF). As polymer molecular weight increases, a clear decrease in the fluorescence intensity is observed.

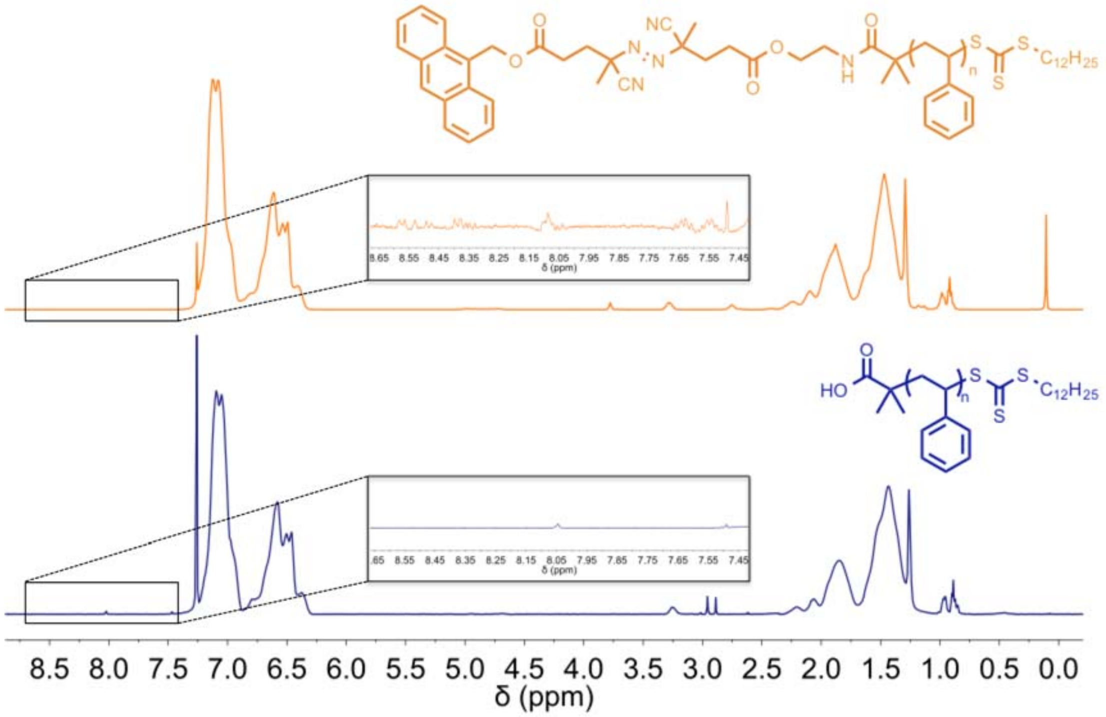


**Fig. S4** $^{1}$H NMR spectra of PS (blue) and PS-Azo-Dye (orange). The appearance of new resonances from 7.50-8.50 ppm indicate the successful attachment of the anthracene dye to the distal terminus of the polymeric spacer.

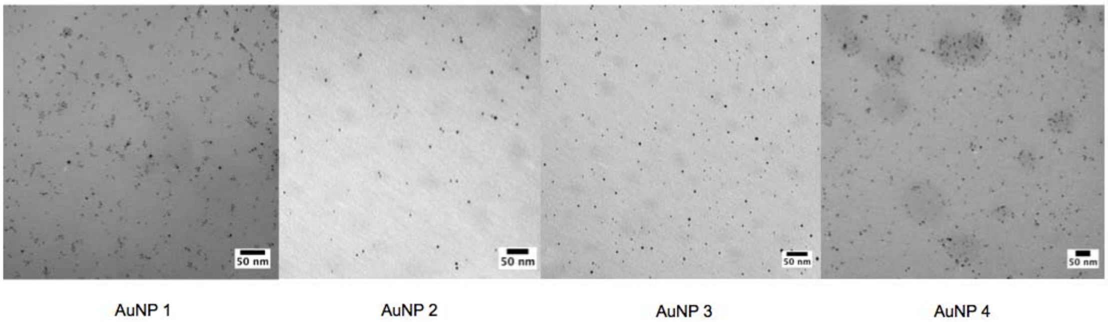


**Fig. S5** Transmission electron micrographs of **AuNP 1-4.** Average diameters are approximately 6 nm.

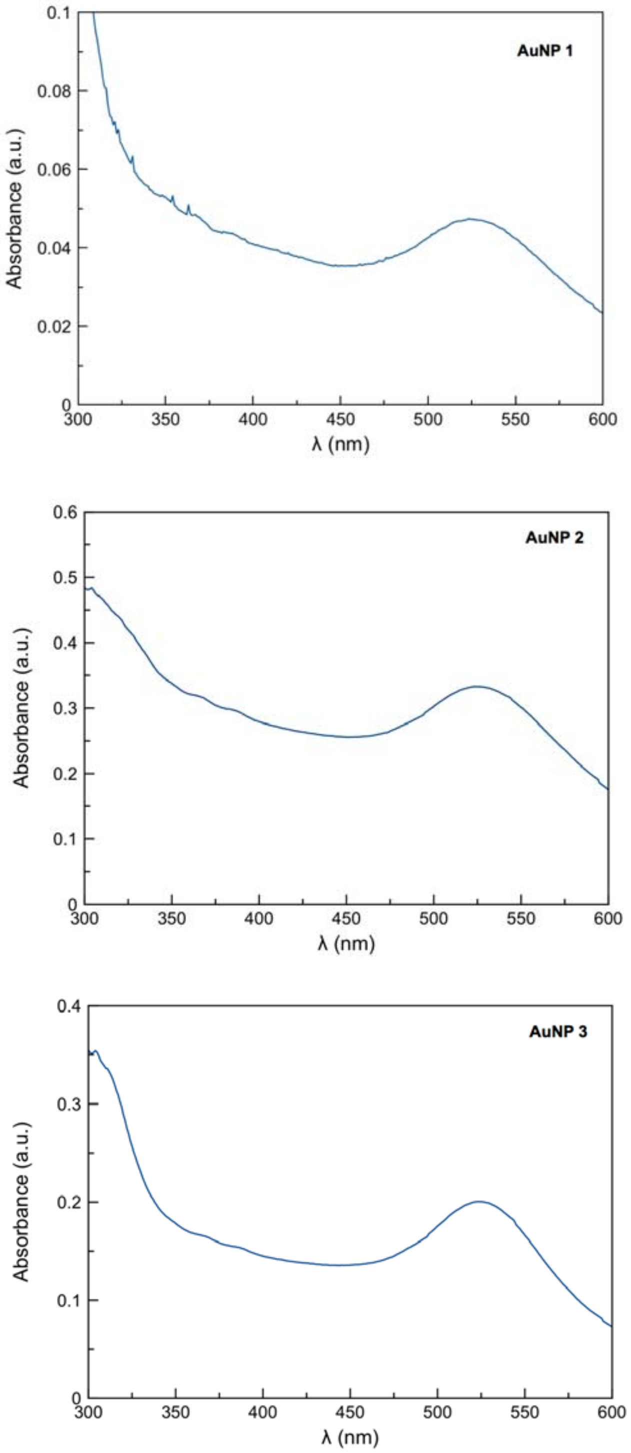


**Fig. S6** Representative UV-Vis spectra of gold nanoparticles. The $\lambda_{max}$ of each nanoparticle sample remains at 520 nm, consistent with their diameters in the range of 6 nm. Moreover, absorption bands of anthracene are visible at 360-380 nm.

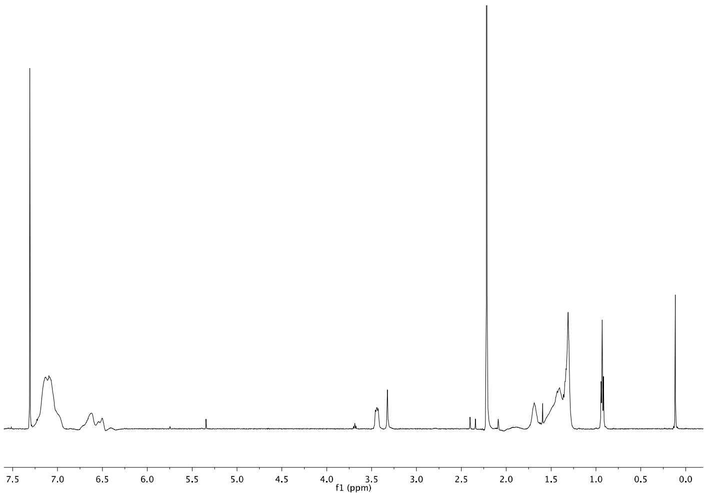


**Fig. S7** $^{1}$H NMR spectrum of polystyrene-stabilized gold nanoparticles. The aromatic and backbone regions of polystyrene are visible at 6.5-7.25 ppm and 1.25-2.0 ppm, respectively.

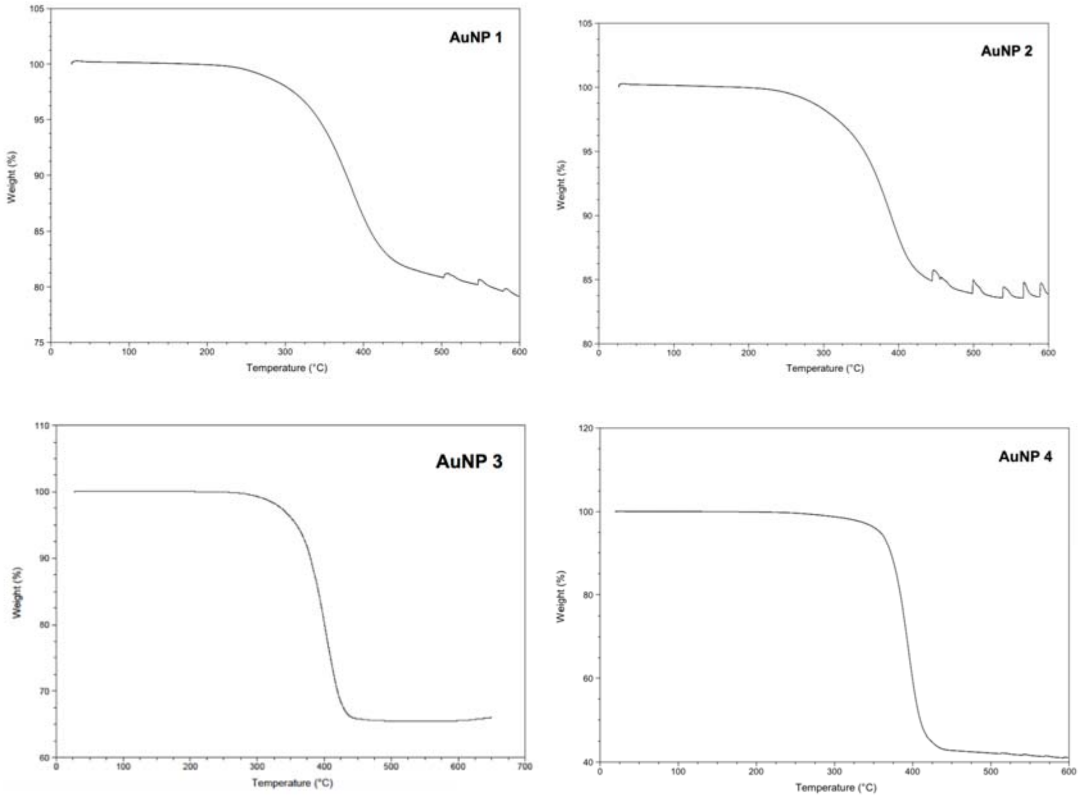


**Fig. S8** Plots of weight loss of polystyrene-stabilized gold nanoparticles as a function of temperature as measured by thermogravimetric analysis. The weight loss from 300-425 °C was attributed to polystyrene, with the remaining percent mass at 650 °C assigned to the gold nanoparticles.

**Table S1.** Values used for ligand density calculations

| | AuNP 1 | AuNP 2 | AuNP 3 | AuNP 4 |
|---|---|---|---|---|
| **PS Mass Loss (%)**[a] | 17.99 | 16.23 | 33.01 | 53.89 |
| **PS MW (kg/mol)** [b] | 2.5 | 6.7 | 17.8 | 34.4 |
| **# of PS Chains ($\times 10^{-16}$)** | 5.42 | 1.56 | 2.36 | 2.27 |
| **AuNP Radius (nm)**[c] | 2.50 | 2.47 | 2.85 | 2.42 |
| **Particle Mass ($\times 10^{18}$ g)**[d] | 1.26 | 1.21 | 1.64 | 1.15 |
| **AuNP Mass Remaining (%)**[e] | 81.75 | 83.66 | 66.99 | 44.91 |
| **# of AuNPs ($\times 10^{-14}$)** | 8.12 | 7.37 | 8.60 | 9.55 |
| **Chains/Particle** | 66.8 | 21.2 | 27.4 | 23.7 |
| **Chains/nm$^2$** | 0.85 | 0.28 | 0.29 | 0.32 |

[a] Measured as weight loss from 300-425 °C by TGA; [b] From GPC-MALLS; [c] Measured by TEM; [d] Calculated from AuNP radius using density of gold (19.3 g/cm$^3$); [e] Percent mass remaining at 600 °C by TGA

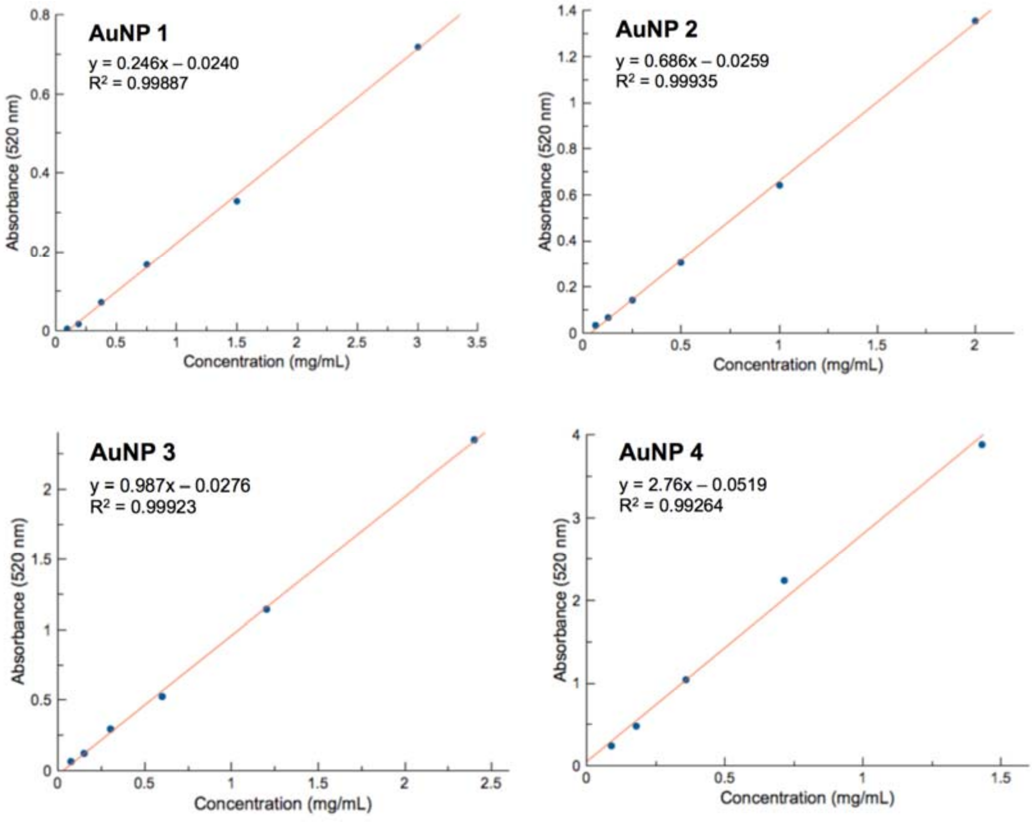


**Fig. S9** UV-Vis calibration curves of "blank" **AuNP 1-4**. The linear fits were used to determine the concentration of functionalized gold nanoparticles in solution.

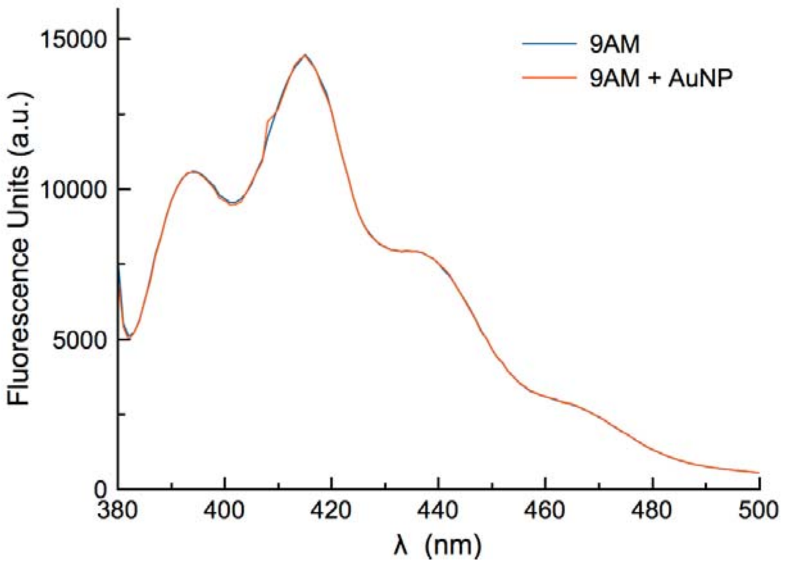


**Fig. S10** Fluorescence spectra of 9-anthracenemethanol (10 µg/mL) in toluene (blue) and 9-anthracenemethanol (10 µg/mL) in the presence of 0.2 mg/mL gold nanoparticles (orange).

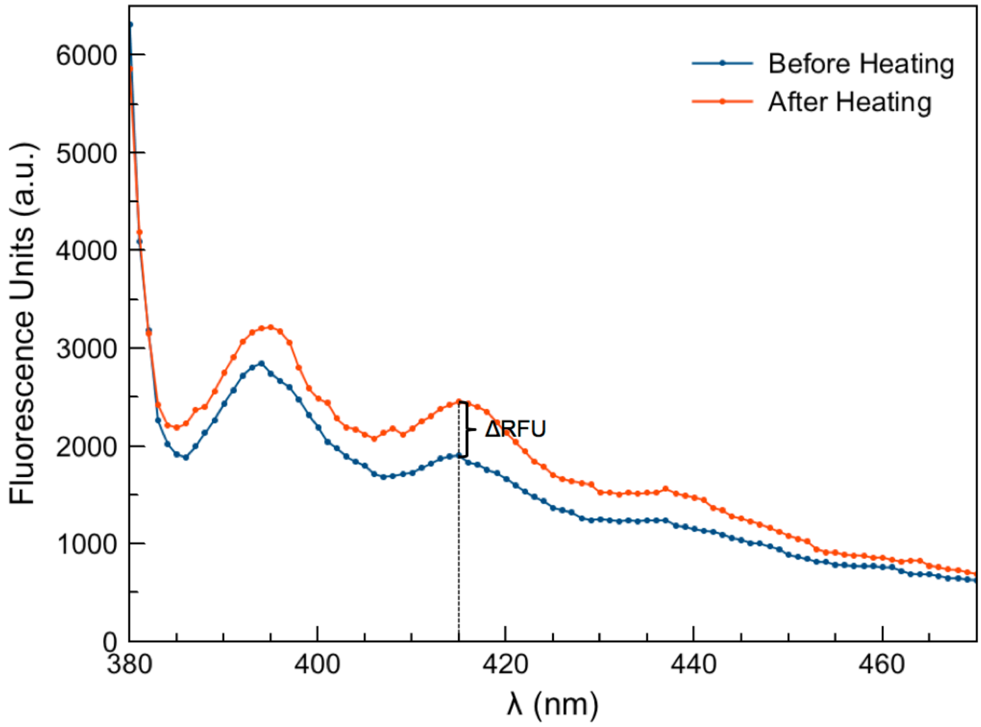


**Fig. S11** Representative fluorescence spectra of an AuNP sample before and after heating at 90 °C for 18 h. The change in fluorescence (ΔRFU) was measured at 415 nm. The same procedure was followed to obtain values to construct normalized fluorescence plots and calibration curves for all samples.

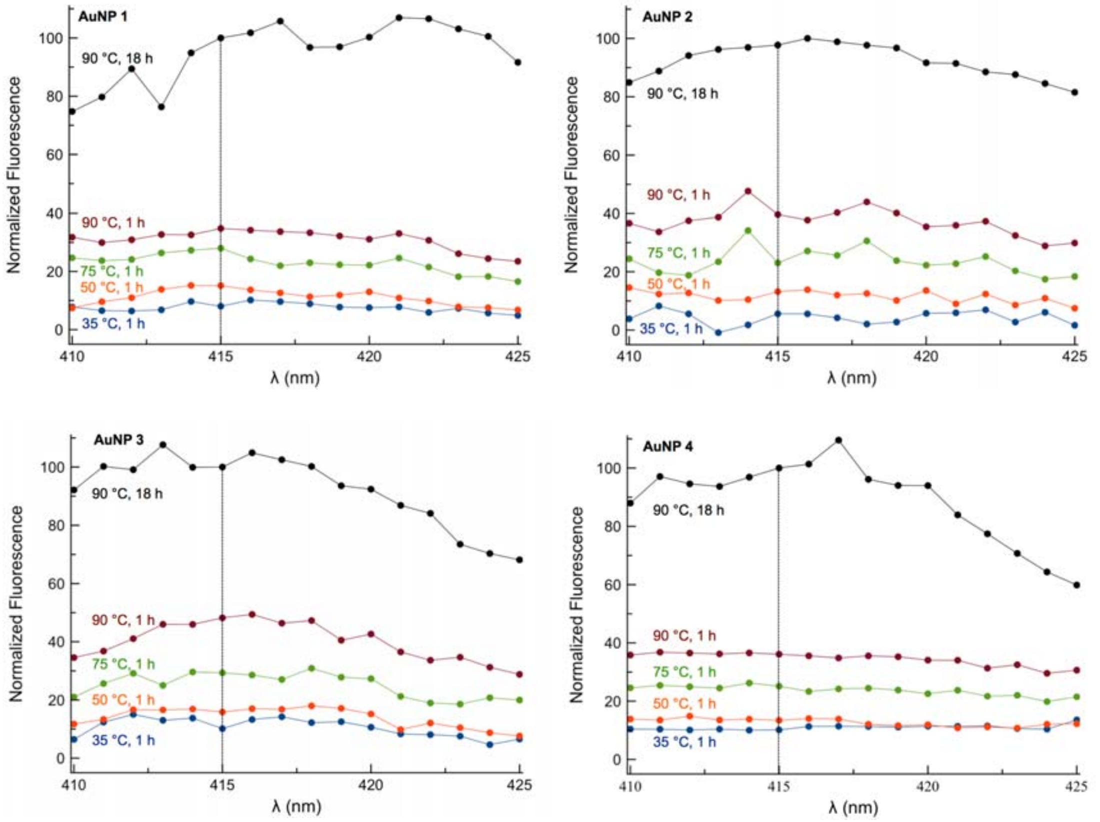


**Fig. S12** Fluorescence signals for **AuNP 1-4** solutions (0.2 mg/mL) in toluene heated at temperatures ranging from 35 to 90 °C for 1 h, normalized to the fluorescence signal obtained after heating at 90 °C for 18 h. The fluorescence intensity at 415 nm was taken as $I_{max}$ and investigated in this study.

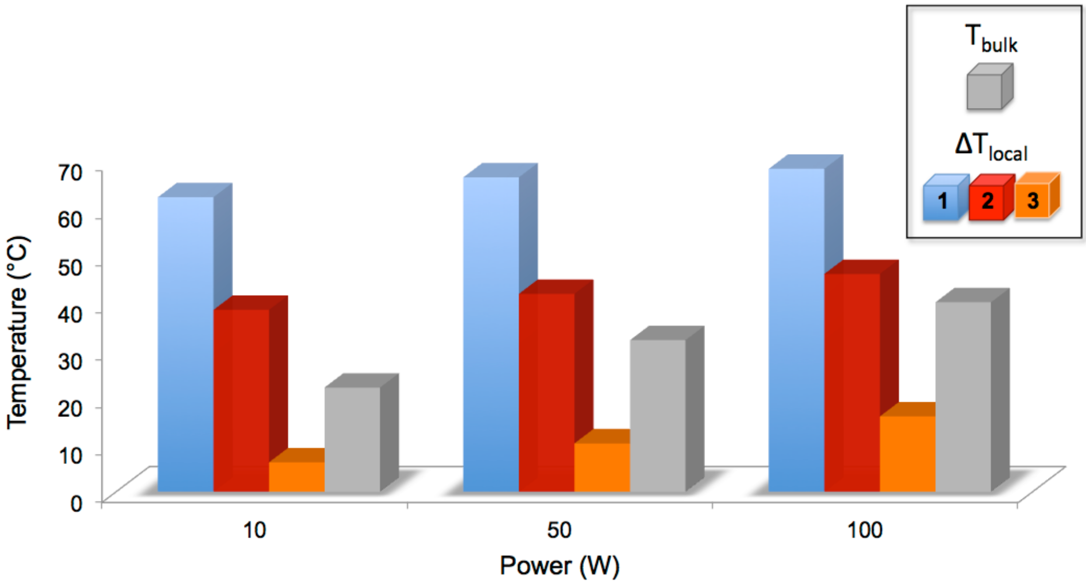


**Fig. S13** Bulk temperature ($T_{bulk}$) and local temperature increase ($\Delta T_{local}$) as a function of power for **AuNP 1-3**. The bulk temperature of the toluene solution increases more rapidly than the localized temperature increase as power is increased.

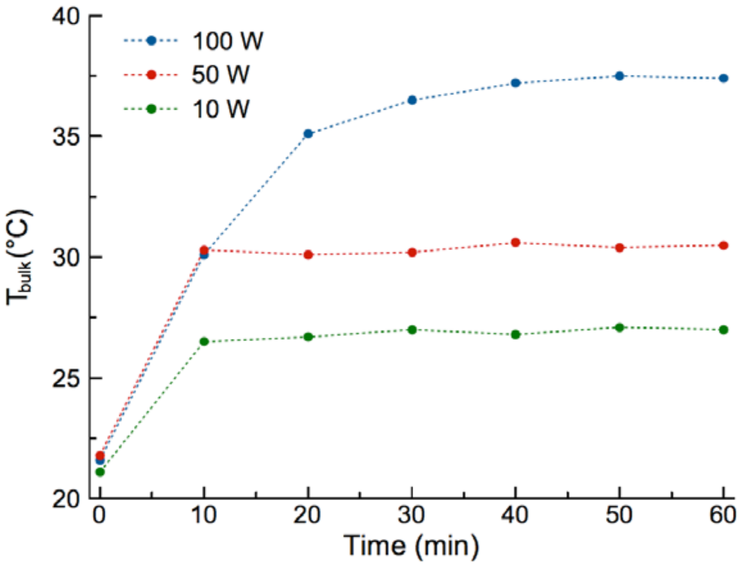


**Fig. S14** Plot of $T_{bulk}$ as a function of time under microwave irradiation (10, 50, and 100 W). The bulk temperature reaches a plateau relatively early in the irradiation time, and remains consistent thereafter.

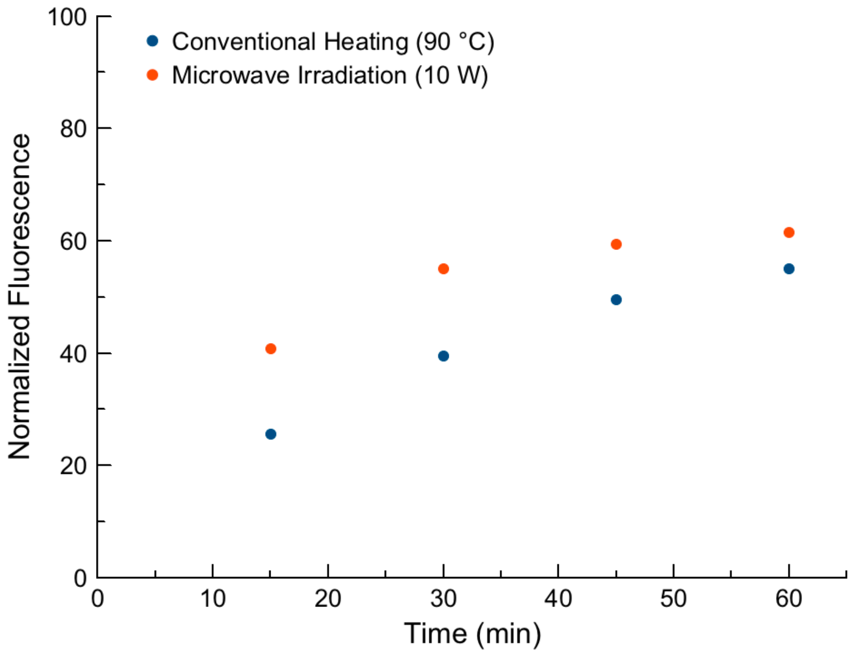


**Fig. S15** Kinetic study on the percent release of the anthracene fluorophore for conventional heating at 90 °C (blue) and microwave irradiation at 10 W (orange). The release kinetics follow a similar exponential decay, indicating that the local temperature remains consistent throughout microwave heating.

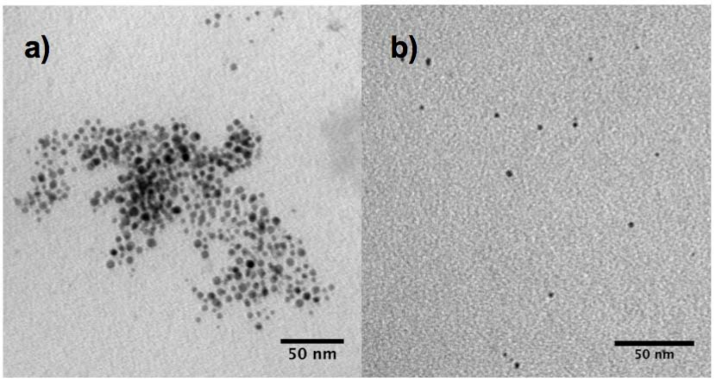


**Fig. S16** Transmission electron micrographs of gold nanoparticles (a) after prolonged storage and (b) following sonication.
